# Effect of diabetes on caregiver burden in an observational study of individuals with Alzheimer’s disease

**DOI:** 10.1186/s12877-016-0264-8

**Published:** 2016-05-03

**Authors:** Jeremie Lebrec, Haya Ascher-Svanum, Yun-Fei Chen, Catherine Reed, Kristin Kahle-Wrobleski, Ann Marie Hake, Joel Raskin, Ebrahim Naderali, Dara Schuster, Robert J. Heine, David M. Kendall

**Affiliations:** Lilly Deutschland GmbH, Bad Homburg, Germany; Eli Lilly and Company, Indianapolis, IN USA; Eli Lilly and Company Limited, Lilly Research Centre, Windlesham, Surrey, UK; Department of Neurology, Indiana University School of Medicine, Indianapolis, IN USA; Eli Lilly and Company, Toronto, Canada; Eli Lilly and Company, Lilly House, Basingstoke, Hampshire UK; Faculty of Science, Liverpool Hope University, Liverpool, UK

**Keywords:** Caregiver burden, Activities of daily living, Supervision, Healthcare resource use, Alzheimer’s disease, Diabetes, Observational study

## Abstract

**Background:**

The burden on caregivers of patients with Alzheimer’s disease (AD) is associated with the patient’s functional status and may also be influenced by chronic comorbid medical conditions, such as diabetes. This *post-hoc* exploratory analysis assessed whether comorbid diabetes in patients with AD affects caregiver burden, and whether caregivers with diabetes experience greater burden than caregivers without diabetes. Caregiver and patient healthcare resource use (HCRU) were also assessed.

**Methods:**

Baseline data from the GERAS observational study of patients with AD and their caregivers (both *n* = 1495) in France, Germany and the UK were analyzed.

Caregiver burden was assessed using the Zarit Burden Interview (ZBI). Caregiver time on activities of daily living (ADL: basic ADL; instrumental ADL, iADL) and supervision (hours/month), and caregiver and patient HCRU (outpatient visits, emergency room visits, nights hospitalized) were assessed using the Resource Utilization in Dementia instrument for the month before the baseline visit. Regression analyses were adjusted for relevant covariates. Time on supervision and basic ADL was analyzed using zero-inflated negative binomial regression.

**Results:**

Caregivers of patients with diabetes (*n* = 188) were younger and more likely to be female (both *p* < 0.05), compared with caregivers of patients without diabetes (*n* = 1307). Analyses showed caregivers of patients with diabetes spent significantly more time on iADL (+16 %; *p* = 0.03; increases were also observed for basic ADL and total caregiver time but did not reach statistical significance) and had a trend towards increased ZBI score. Patients with diabetes had a 63 % increase in the odds of requiring supervision versus those without diabetes (*p* = 0.01). Caregiver and patient HCRU did not differ according to patient diabetes.

Caregivers with diabetes (*n* = 127) did not differ from those without diabetes (*n* = 1367) regarding burden/time, but caregivers with diabetes had a 91 % increase in the odds of having outpatient visits (*p* = 0.01).

**Conclusions:**

This cross-sectional analysis found caregiver time on iADL and supervision was higher for caregivers of patients with AD and diabetes versus without diabetes, while HCRU was unaffected by patient diabetes. Longitudinal analyses assessing change in caregiver burden over time by patient diabetes status may help clarify the cumulative impact of diabetes and AD dementia on caregiver burden.

## Background

Alzheimer’s disease (AD) is the most common cause of dementia, accounting for 60–80 % of dementia cases, and prevalence increases with age [1]. In 2012, dementia was estimated to affect 8.4 million people aged ≥60 years in the European Union (EU), approximately 7 % of this age group [1]. Although incidence rates vary, AD prevalence is generally consistent between European countries and the USA [2].

People with AD have been reported to be at increased risk of developing diabetes [3], and people with diabetes are at increased risk of developing cognitive impairment and AD dementia [4–7]. Around 60 million people in Europe, ~8–10 % of the adult population, have diabetes or impaired glucose tolerance [8, 9]. Estimates of the prevalence of diabetes in people with dementia range between 6 and 39 % [10], although most studies reviewed by Bunn et al. [10] found no increase in diabetes prevalence for people with versus without dementia (one study suggested a lower prevalence of diabetes in hospitalized people with dementia [11]). The risk of diabetes increases with longevity [8, 9]; recent US data suggest a diabetes prevalence of 26 % (diagnosed and undiagnosed) in people aged 65 years and over [12]. Predictions of increasing prevalence of both dementia [13] and diabetes [8, 9, 14] are therefore based on a future aging population.

In addition to shared demographics (e.g., aging, comorbidities, and genetic factors), there are reports of common pathologies that link AD and diabetes, such as amyloid deposits, cardiovascular risk factors, inflammation, glucose toxicity, and changes in insulin metabolism [6, 15–17]. Although cardiovascular risk factors are more pathophysiologically relevant to vascular dementia than AD, they can affect the rate of cognitive decline in AD dementia [6].

As AD dementia and diabetes are both chronic age-related diseases, caregivers of affected patients experience substantial burden [18, 19]. Burden may be influenced by several factors, and can include both objective burden, such as the amount of time spent helping the patient, and the subjective burden perceived by the caregiver, as measured using the Zarit Burden Interview (ZBI; [20]).

In AD dementia, caregiver burden is associated with the functional status of the patient [21] and may also be related to chronic comorbid medical conditions, such as diabetes. A small focus group study with 21 caregivers of patients with dementia and type 2 diabetes reported that caring for patients with both conditions is associated with a high level of caregiver burden [22]. However, there are contrasting reports about the weight of the burden on those caring for patients with AD dementia or diabetes among elderly populations. While diabetes was not shown to be an independent predictor of burden in caregivers of community-dwelling frail elderly patients [23], a study by Langa and colleagues reported diabetes as a factor in increasing caregiver burden [24]. Dementia patient comorbidities and dependence are also associated with increased caregiver healthcare resource use (HCRU) and costs [25].

Caregiver burden and HCRU may also be related to the health and functional status of the caregiver; caregivers are often of a similar age to the patient and have various medical conditions. Caregivers with medical conditions of their own, such as diabetes, may experience greater burden than those without such conditions, due to the added responsibility of caring for the patient with AD dementia alongside managing their own treatment. Differences in social support given to caregivers may also affect caregiver time and perceived burden [26]. Identifying factors that influence burden and HCRU may help to inform possible support measures for patients and caregivers.

GERAS is a prospective observational study of community-dwelling patients with AD and their informal caregivers in three European countries: France, Germany, and the UK [27]. This *post-hoc* exploratory analysis assessed whether caregiver burden among GERAS participants was affected by the presence of comorbid diabetes in patients with AD, and whether caregivers with diabetes experienced greater burden than caregivers without diabetes. The effect of diabetes status on caregiver and patient HCRU was also evaluated.

## Methods

### Study design

GERAS is a large 18-month multicenter, prospective observational study designed to assess the costs and resource use associated with AD for patients and their primary caregivers in France, Germany, and the UK. Full details of the study design and baseline findings [27], and an assessment of the effects of AD dementia progression on costs and caregiver-related outcomes at 18 months [28], have been reported previously. The present analysis was restricted to data from the baseline visit.

The study included community-dwelling patients aged ≥55 years, presenting during the normal course of care, diagnosed with probable AD according to the National Institute of Neurological and Communicative Disorders and Stroke and Related Disorders Association criteria [29], and with a Mini-Mental State Examination (MMSE) score [30] of ≤26 points. Each patient also had to have an informal (i.e., non-professional) caregiver who was willing to take responsibility for the patient for at least 6 months of the year (primary caregiver) and to participate in the study. Ethical review board approval of the study was obtained in each country according to individual country regulations. Written informed consent was obtained from both the patient (or the patient’s legal representative; patients were asked to give at least co-consent where able) and caregiver.

Patients were stratified according to AD dementia severity at baseline as reported previously [27]: mild (MMSE score 21–26 points), moderate (MMSE 15–20 points), or moderately severe/severe AD dementia (MMSE <15 points). The GERAS study aimed to recruit a minimum of 600 patients in each country (in similar proportions of 200 in each severity group) to obtain a 95 % confidence interval (CI) of ±10 % of the mean cost estimate (based on the assumptions described in Wimo et al. [27]). The primary analysis reported here is based on the total AD dementia severity population (MMSE ≤26 points). Secondary analyses were performed based on the subgroup with mild/moderate AD dementia (MMSE 16–26 points; consistent with the MMSE criteria for patients included in the first two phase 3 studies of solanezumab treatment [31]).

Patients enrolled in GERAS and their caregivers were both queried at baseline regarding whether they suffered from a list of common medical conditions, including diabetes (information regarding patient medical conditions could be provided by the patient or caregiver). For this study, anyone positively endorsing “diabetes” was considered to have diabetes. Apart from whether or not diabetes medication was being received (Yes/No), no further diabetes-related information, e.g., regarding diabetes or medication type, or glycemic control, was requested.

### Measures of caregiver burden

Both subjective and objective measures of burden were considered in this analysis. Subjective caregiver burden was assessed using the shortened 22-item version of the original 29-item ZBI [20]. The ZBI provides a recognized measure of the amount of subjective burden perceived by the caregiver and is a valid, reliable and widely recognized measure of caregiver burden [21, 32]. It is a self-report inventory administered to the caregiver during an assessment interview. Caregivers are asked to rate their feelings regarding their health and psychological wellbeing, finances, social life, and relationship with the patient. Responses are used to derive the ZBI total score (score range 0–88), where higher scores represent greater burden.

Objective burden, i.e., caregiver time spent looking after the patient, was assessed using the Resource Utilization in Dementia (RUD) instrument [33], version RUD Complete 3.1, by interview with the caregiver. This is a widely used standardized instrument for collecting resource use data in dementia and has been validated for use in different care settings, including community-dwelling patients [34, 35]. Time (in the month before the baseline visit) was recorded as the total number of caregiving hours, including the number of hours spent on basic ADL (e.g., help with eating, getting dressed), instrumental ADL (iADL; e.g., cooking, shopping), and patient supervision (i.e., watching the patient to prevent dangerous events).

### Measures of HCRU

Patient and caregiver HCRU (number of outpatient visits, emergency room [ER; accident and emergency department] visits, and nights hospitalized) was also assessed using the RUD instrument [33] for the month before the baseline visit. HCRU could be for any purpose and may not have been related to AD dementia or diabetes.

### Statistical analysis

Baseline characteristics of caregivers and patients were summarized based on non-missing observations. Data are presented as mean (standard deviation [SD]) or as numbers and percentages of caregivers/patients. The *p*-values presented for comparisons of baseline characteristics between patient/caregiver diabetes groups were obtained using Analysis of Variance (ANOVA) for continuous variables, with country and diabetes status as factors; and Cochran–Mantel–Haenszel test for categorical variables, with stratification by country.

Descriptive statistics for caregiver burden (ZBI) are presented as mean (SD), caregiver time measures as median with interquartile range (Q1–Q3), and HCRU as n (%) of caregivers/patients using each type of healthcare resource (i.e., outpatient or ER visits, nights hospitalized).

In order to investigate the possible causal effect of diabetes status on outcomes of interest, a propensity score approach was adopted to adjust for any potential imbalance of the core baseline data between diabetes and non-diabetes groups. Propensity scores for patient and caregiver diabetes status were derived separately based on age, body mass index (BMI), and sex for patients, and on age and sex for caregivers (ethnicity and caregiver BMI were not collected in GERAS).

Analyses were performed using regression models adjusted for the following covariates to minimize confounding: country, age, sex, AD dementia severity at baseline (mild, moderate, moderately severe/severe), patient BMI, concomitant use of acetylcholinesterase inhibitors, concomitant use of memantine, education (<8 years, 8–12 years, >12 years), time since AD diagnosis, whether the caregiver was living with the patient (no vs. yes), patient living location (rural vs. urban), living arrangement (living alone; not living alone + married; not living alone + not married), and propensity score.

The effect of diabetes status on ZBI score at baseline was assessed using Analysis of Covariance (ANCOVA). Effect of diabetes on caregiver time was assessed using a generalized linear model with gamma distribution and log link. Due to a high number of zero values (i.e., as many patients do not require help with basic ADL or supervision), the number of hours spent on basic ADL and supervision time were analyzed using zero-inflated negative binomial regression models. HCRU variables were analyzed as dichotomized endpoints using logistic regression.

Results from analyses are presented as absolute difference, percentage increase, or percentage increase based on odds ratios with *p*-values and 95 % CIs for patients/caregivers with versus without diabetes. The *p*-values presented for comparisons between patient/caregiver diabetes groups were obtained using likelihood ratio statistics for all measures except requirement for supervision and caregiver time on basic ADL, where only the Wald test was available.

To assess the effect of AD dementia severity, secondary analyses were conducted using data from the subgroup with mild/moderate AD dementia (MMSE score 16–26 points).

Sensitivity analyses were performed to assess the effect of excluding propensity scores and patient BMI (due to the high level of missing data for BMI) from the main analyses.

All data were analyzed using SAS software Version 9.2 (SAS Institute, Cary, North Carolina, USA).

## Results

### Comparison: patients with diabetes versus without diabetes

Of the 1495 patients with AD included in this study, 188 (12.6 %) had diabetes. Most patients with diabetes (87.8 %) were receiving diabetes medication. Patients with and without diabetes did not differ significantly in time since AD diagnosis, MMSE score and AD medication use (Table 1). Patients with diabetes were less likely to be married/cohabiting, more likely to be living alone in their own home, and had fewer years of education than patients without diabetes (all *p* < 0.05). Patients with diabetes had a slightly but significantly higher BMI (mean 26.3 vs. 25.2 kg/m^2^, *p* < 0.001) than patients without diabetes.

**Table 1:** Patient and caregiver characteristics according to diabetes status of the patient with AD dementia

Characteristic^a^	Overall *N* = 1495	Patients with diabetes *N* = 188	Patients without diabetes *N* = 1307	*P*-value
Patients				
Age, years^b^	77.6 (7.65)	78.0 (6.97)	77.5 (7.74)	0.137
Sex, n (%) female	819 (54.8)	94 (50.0)	725 (55.5)	0.171
BMI, kg/m^2^	25.3 (4.07)	26.3 (4.20)	25.2 (4.02)	**<0.001**
Marital status, n (%)				**0.019**
Married/cohabiting	1076 (72.0)	122 (64.9)	954 (73.0)	
Widowed	361 (24.2)	60 (31.9)	301 (23.0)	
Divorced/separated	36 (2.4)	2 (1.1)	34 (2.6)	
Never married	21 (1.4)	4 (2.1)	17 (1.3)	
Living location, n (%)				0.892
Urban	1131 (75.7)	143 (76.1)	988 (95.7)	
Rural	363 (24.3)	45 (23.9)	318 (24.3)	
Living in own home, n (%)	1428 (95.7)	179 (95.7)	1249 (95.7)	0.220
Living alone in own home, n (%)	253 (17.7)	33 (18.4)	220 (17.6)	**0.017**
Alcohol consumption, n (%)	885 (61.4)	91 (51.1)	794 (62.9)	**0.043**
Years of education	10.4 (3.15)	9.5 (2.84)	10.6 (3.17)	**<0.001**
Time since AD diagnosis, years	2.2 (2.23)	2.1 (2.00)	2.3 (2.26)	0.268
Patients with comorbidities^c^, n (%)	1101 (73.6)	188 (100)	913 (69.9)	0.144
Diabetes drug use, n (%)		165 (87.8)		
Experienced a fall in last 3 months, n (%)	196 (13.1)	26 (13.8)	170 (13.0)	0.465
MMSE total score (range 0–30)	17.4 (6.34)	17.4 (6.14)	17.4 (6.37)	0.909
Neuropsychological assessment in last 6 months, n (%)	841 (56.4)	108 (57.4)	733 (56.2)	0.499
AD drug use, n (%)				0.750
No AD medications	212 (14.2)	29 (15.4)	183 (14.0)	
One AD medication	1121 (75.0)	141 (75.0)	980 (75.0)	
Two or more AD medications	162 (10.8)	18 (9.6)	144 (11.0)	
Psychiatric/hypnotic drug use, n (%)	393 (26.3)	44 (23.4)	349 (26.7)	0.656
Caregivers				
Age, years^b^	67.3 (12.04)	65.0 (12.69)	67.7 (11.91)	**0.012**
Sex, n (%) female	958 (64.2)	139 (73.9)	819 (62.8)	**0.005**
Marital status, n (%)				0.403
Married/cohabiting	1316 (88.1)	163 (86.7)	1153 (88.4)	
Widowed	38 (2.5)	3 (1.6)	35 (2.7)	
Divorced/separated	70 (4.7)	9 (4.8)	61 (4.7)	
Never married	69 (4.6)	13 (6.9)	56 (4.3)	
Relationship to the patient, n (%)				**0.001**
Wife	503 (33.7)	70 (37.2)	433 (33.2)	
Husband	481 (32.2)	39 (20.7)	442 (33.9)	
Child	405 (27.1)	69 (36.7)	336 (25.7)	
Friend	20 (1.3)	2 (1.1)	18 (1.4)	
Other	84 (5.6)	8 (4.3)	76 (5.8)	
Caregiver lives with the patient, n (%)	1135 (76.0)	141 (75.0)	994 (76.2)	0.781
Caregivers with medical conditions^c^, n (%)	875 (58.6)	105 (55.9)	770 (59.0)	0.181

*AD* Alzheimer’s disease, *BMI* body mass index, *MMSE* Mini-Mental State Examination

^a^Data are presented as mean (SD) unless indicated otherwise. Percentages reported are for patients/caregivers with data available for each specific variable. Missing data for patients (overall): no missing data for age, sex, comorbidities, diabetes drug use, MMSE total score, AD drug use, psychiatric/hypnotic drug use; amount of missing data for other variables were BMI (*n* = 220), marital status (*n* = 1), living location (*n* = 1), living in own home (*n* = 3), living alone in own home (*n* = 67), alcohol consumption (*n* = 54), years of education (*n* = 6), time since AD diagnosis (*n* = 1), experienced a fall (*n* = 2), neuropsychological assessment (*n* = 3). Missing data for caregivers (overall) was *n* = 2 for all variables except medical conditions (*n* = 1; caregiver diabetes status not collected)

^b^The Case Report Form collected only birth year data, thus the missing month and day were imputed with 15 July. Age is the year part of the difference between the date of Informed Consent and the imputed birth date

^c^Data include the presence of diabetes

*P*-values are for the comparison between patient diabetes groups (ANOVA for continuous variables, with country and diabetes status as factors, and Cochran-Mantel-Haenszel test for categorical variables, with stratification by country)

*P*-values indicating statistically significant differences between patient diabetes groups (*p* < 0.05) are highlighted in bold type

#### Caregiver baseline characteristics

Caregivers of patients with diabetes were younger (mean age 65.0 vs. 67.7 years, *p* = 0.012) and more likely to be female (73.9 % vs. 62.8 %, *p* = 0.005) and the patient’s wife (37.2 % vs. 33.2 %) or adult child (36.7 % vs. 25.7 %; *p* = 0.001 for caregiver relationship to the patient), compared with caregivers of patients without diabetes (Table 1).

#### Caregiver burden

The overall mean (SD) ZBI total score at baseline was 29.0 (15.07); Table 2. All caregiver time measures showed wide variation within the patient diabetes groups (Table 2); for example, although an overall median of 60.0 h was reported for iADL in the month before baseline, the interquartile range (Q1–Q3) was 20.0–120.0 h. Analyses were adjusted for potentially confounding covariates.

**Table 2:** Caregiver burden/time, and caregiver and patient HCRU according to patient diabetes status (descriptive statistics)

Total AD dementia severity population				Mild/moderate AD dementia		
Parameter	Overall *N* = 1495	Patients with diabetes *N* = 188	Patients without diabetes *N* = 1307	Overall *N* = 985	Patients with diabetes *N* = 119	Patients without diabetes *N* = 866
Caregivers						
ZBI score, mean (SD)	29.0 (15.07)	30.8 (15.37)	28.8 (15.01)	26.6 (14.71)	28.1 (14.81)	26.4 (14.69)
Total caregiver time (hours), median (Q1-Q3)	105.0 (30.0–330.0)	122.5 (45.0–415.0)	105.0 (30.0–322.0)	75.0 (20.0–180.0)	84.0 (21.0–194.0)	70.0 (20.0–180.0)
Hours on basic ADL, median (Q1-Q3)	6.0 (0.0–60.0)	15.0 (0.0–60.0)	5.0 (0.0–60.0)	0.0 (0.0–30.0)	0.0 (0.0–24.0)	0.0 (0.0–30.0)
Hours on iADL, median (Q1-Q3)	60.0 (20.0–120.0)	60.0 (22.5–120.0)	60.0 (20.0–120.0)	46.5 (15.0–90.0)	56.0 (15.0–90.0)	45.0 (14.0–90.0)
Hours on supervision, median (Q1-Q3)	8.0 (0.0–120.0)	30.0 (0.0–206.0)	4.0 (0.0–104.5)	0.0 (0.0–30.0)	0.0 (0.0–60.0)	0.0 (0.0–30.0)
Caregiver outpatient visits, n (%)						
No visits	636 (42.6)	80 (42.6)	556 (42.6)	410 (41.7)	46 (38.7)	364 (42.1)
1 visit	401 (26.9)	45 (23.9)	356 (27.3)	270 (27.4)	31 (26.1)	239 (27.6)
2 visits	199 (13.3)	22 (11.7)	177 (13.6)	134 (13.6)	15 (12.6)	119 (13.8)
3 visits	97 (6.5)	14 (7.4)	83 (6.4)	62 (6.3)	9 (7.6)	53 (6.1)
>3 visits	160 (10.7)	27 (14.4)	133 (10.2)	108 (11.0)	18 (15.1)	90 (10.4)
Caregivers with ≥1 ER visit in past month, n (%)	45 (3.0)	2 (1.1)	43 (3.3)	28 (2.8)	1 (0.8)	27 (3.1)
Caregivers with ≥1 night hospitalization in past month, n (%)	34 (2.3)	2 (1.1)	32 (2.5)	24 (2.4)	2 (1.7)	22 (2.5)
Patients						
Patient outpatient visits, n (%)						
No visits	289 (19.4)	26 (13.8)	263 (20.2)	185 (18.8)	19 (16.0)	166 (19.2)
1 visit	415 (27.8)	43 (22.9)	372 (28.5)	275 (27.9)	26 (21.8)	249 (28.8)
2 visits	315 (21.1)	41 (21.8)	274 (21.0)	211 (21.4)	22 (18.5)	189 (21.8)
3 visits	134 (9.0)	22 (11.7)	112 (8.6)	90 (9.1)	13 (10.9)	77 (8.9)
>3 visits	340 (22.8)	56 (29.8)	284 (21.8)	223 (22.7)	39 (32.8)	184 (21.3)
Patients with ≥1 ER visit in past month, n (%)	43 (2.9)	7 (3.7)	36 (2.8)	24 (2.4)	4 (3.4)	20 (2.3)
Patients with ≥1 night hospitalization in past month, n (%)	62 (4.2)	14 (7.4)	48 (3.7)	36 (3.7)	7 (5.9)	29 (3.4)

*AD* Alzheimer’s disease, *ADL* activities of daily living, *ER* emergency room, *HCRU* health care resource use, *iADL* instrumental ADL, *Q* quartile, *SD* standard deviation, *ZBI* Zarit Burden Interview

Percentages reported are for caregivers and patients with data available for each specific variable. Missing data for the total AD dementia population (overall diabetes status) were *n* = 2 for all measures (for caregivers and patients) except ZBI score (*n* = 10). Missing data based on the population with mild/moderate AD dementia were *n* = 1 for all measures (for caregivers and patients) except ZBI score (*n* = 7)

**Fig. 1:**
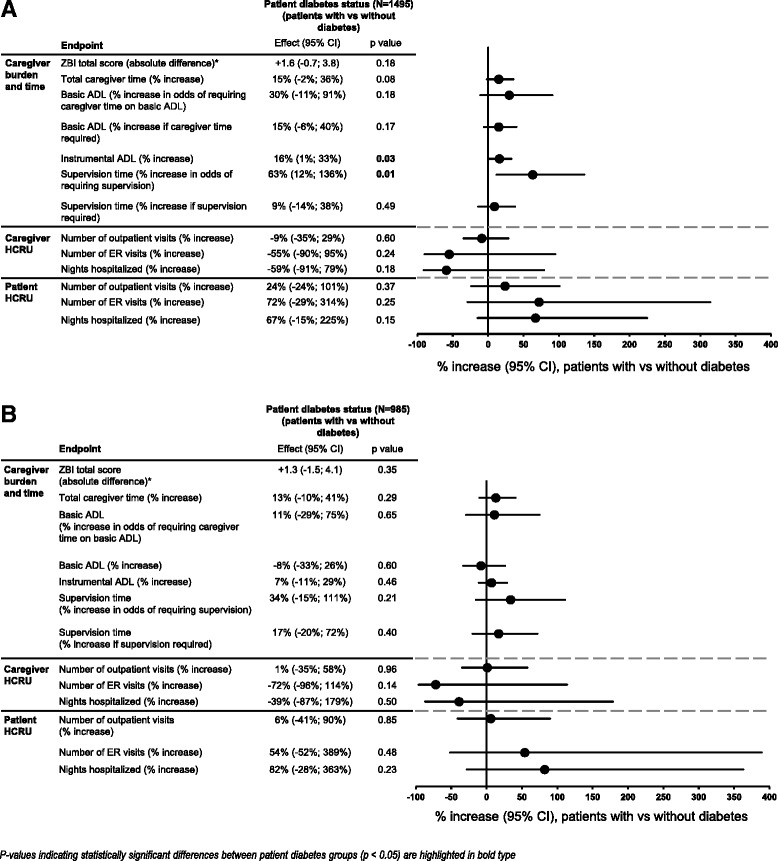
Caregiver burden/time, and caregiver and patient HCRU according to patient diabetes status. **a** Total AD dementia population. **b** Mild/moderate AD dementia population. AD Alzheimer’s disease, ADL activities of daily living, CI confidence interval, ER emergency room, HCRU health care resource use, ZBI Zarit Burden Interview. Data are presented as absolute difference or % increase with 95 % CIs for patients with versus without diabetes. HCRU measures are reported as the percentage increase in the odds of having outpatient or ER visits or of being hospitalized. *P*-values (obtained using likelihood ratio statistics for all measures except requirement for supervision and caregiver time on basic ADL, where only the Wald test was available) are for the comparison between patient diabetes groups. *As ZBI data are presented as absolute difference it was not possible to include this measure in the Forest plot with the effects of the other endpoints, which are presented as % increase

Figure 1a shows a consistent general effect of increased burden/time requirement for caregivers of patients with versus without diabetes in the main analysis; some of these differences were statistically significant. A trend towards increased ZBI total score was observed for caregivers of patients with diabetes, along with significantly more caregiver time spent on iADL (+16 %; *p* = 0.03). Total caregiver time also showed a similar increase for caregivers of patients with diabetes, although this difference did not reach statistical significance (Fig. 1a). Many patients with AD dementia, particularly those with milder severity, do not require supervision or any caregiver time spent on basic ADL (zero hours for those two outcomes were reported for more than 25 % of patients overall; Table 2). Patients with diabetes had a 63 % increase in the odds of requiring supervision compared with patients without diabetes (*p* = 0.01), although among those who did require supervision there was no significant difference in supervision time for patients with versus without diabetes. The odds of requiring caregiver time helping with basic ADL, and the number of hours spent on basic ADL (for those who required this help), were also higher for patients with versus without diabetes, but these differences did not reach statistical significance (Fig. 1a).

#### Caregiver and patient HCRU

Outpatient visits were more frequently used by patients than caregivers: 1204 patients (80.6 %) and 857 caregivers (57.4 %) overall had one or more outpatient visit in the month before baseline (Table 2). Less than 5 % of caregivers and patients overall had ER visits or hospitalizations during this time (Table 2). No HCRU measures were significantly affected by patient diabetes status (Fig. 1a).

### Comparison: caregivers with versus without diabetes

Overall, 127 caregivers in this analysis had diabetes (8.5 % of the caregiver population), most of whom (92.9 %) were receiving diabetes medication.

#### Caregiver baseline characteristics

Compared with caregivers without diabetes, caregivers with diabetes were older (mean age 72.4 vs. 66.9 years, *p* < 0.001), less likely to be female (54.3 % vs. 65.1 %, *p* = 0.012), and more likely to be the patient’s husband (41.7 % vs. 31.3 %, *p* < 0.001 for caregiver relationship to the patient) and living with the patient (89.0 % vs. 74.8 %, *p* < 0.001); Table 3.

**Table 3:** Caregiver characteristics according to caregiver diabetes status

Characteristic^a^	Overall *N* = 1494^b^	Caregivers with diabetes *N* = 127	Caregivers without diabetes *N* = 1367	*P*-value
Caregiver characteristics				
Age, years^c^	67.3 (12.04)	72.4 (9.67)	66.9 (12.13)	**<0.001**
Sex, n (%) female	958 (64.2)	69 (54.3)	889 (65.1)	**0.012**
Marital status, n (%)				**0.022**
Married/cohabiting	1316 (88.1)	123 (96.9)	1193 (87.3)	
Widowed	38 (2.5)	1 (0.8)	37 (2.7)	
Divorced/separated	70 (4.7)	1 (0.8)	69 (5.1)	
Never married	69 (4.6)	2 (1.6)	67 (4.9)	
Relationship to the patient, n (%)				**<0.001**
Wife	503 (33.7)	50 (39.4)	453 (33.2)	
Husband	481 (32.2)	53 (41.7)	428 (31.3)	
Child	405 (27.1)	13 (10.2)	392 (28.7)	
Friend	20 (1.3)	2 (1.6)	18 (1.3)	
Other	84 (5.6)	9 (7.1)	75 (5.5)	
Caregiver lives with the patient, n (%)	1135 (76.0)	113 (89.0)	1022 (74.8)	**<0.001**
Caregivers with medical conditions^d^, n (%)	127 (100)	748 (54.7)	875 (58.6)	0.178
Diabetes drug use, n (%)		118 (92.9)		

^a^Data are presented as mean (SD) unless indicated otherwise. Percentages reported are for caregivers with data available for each specific variable. Missing data (overall) were *n* = 1 for all variables, except medical conditions and diabetes drug use (no missing data)

^b^Diabetes status was unknown for one caregiver, thus *N* = 1494

^c^The Case Report Form collected only birth year data, thus the missing month and day were imputed with 15 July. Age is the year part of the difference between the date of Informed Consent and the imputed birth date

^d^Data include the presence of diabetes

*P*-values are for the comparison between caregiver diabetes groups (ANOVA for continuous variables, with country and diabetes status as factors, and Cochran–Mantel–Haenszel test for categorical variables, with stratification by country)

*P*-values indicating statistically significant differences between caregiver diabetes groups (*p* < 0.05) are highlighted in bold type

#### Caregiver burden

Again, results for all burden measures were highly variable, both overall and within the different caregiver diabetes groups (Table 4).

**Table 4:** Caregiver burden, time, and HCRU according to caregiver diabetes status (descriptive statistics)

Parameter^a^	Total AD dementia severity population			Mild/moderate AD dementia		
	Overall *N* = 1494^b^	Caregivers with diabetes *N* = 127	Caregivers without diabetes *N* = 1367	Overall *N* = 985	Caregivers with diabetes *N* = 88	Caregivers without diabetes *N* = 897
ZBI score	29.0 (15.07)	26.7 (14.82)	29.2 (15.08)	26.6 (14.71)	24.8 (14.29)	26.8 (14.74)
Total caregiver time, hours	105.0 (30.0–330.0)	120.0 (31.0–375.0)	105.0 (30.0–330.0)	75.0 (20.0–180.0)	82.5 (30.0–210.0)	75.0 (18.0–180.0)
Hours on basic ADL	6.0 (0.0–60.0)	0.0 (0.0–60.0)	7.3 (0.0–60.0)	0.0 (0.0–30.0)	0.0 (0.0–15.0)	0.0 (0.0–30.0)
Hours on iADL	60.0 (20.0–120.0)	60.0 (30.0–120.0)	60.0 (20.0–120.0)	46.5 (15.0–90.0)	48.8 (15.5–90.0)	46.5 (15.0–90.0)
Hours on supervision	8.0 (0.0–120.0)	15.0 (0.0–240.0)	8.0 (0.0–120.0)	0.0 (0.0–30.0)	1.3 (0.0–60.0)	0.0 (0.0–30.0)
Caregiver outpatient visits, n (%)						
No visits	636 (42.6)	35 (27.6)	601 (44.0)	410 (41.7)	24 (27.3)	386 (43.1)
1 visit	401 (26.9)	29 (22.8)	372 (27.2)	270 (27.4)	24 (27.3)	246 (27.5)
2 visits	199 (13.3)	34 (26.8)	165 (12.1)	134 (13.6)	22 (25.0)	112 (12.5)
3 visits	97 (6.5)	10 (7.9)	87 (6.4)	62 (6.3)	7 (8.0)	55 (6.1)
>3 visits	160 (10.7)	19 (15.0)	141 (10.3)	108 (11.0)	11 (12.5)	97 (10.8)
Caregivers with ≥1 ER visit in past month, n (%)	45 (3.0)	3 (2.4)	42 (3.1)	28 (2.8)	2 (2.3)	26 (2.9)
Caregivers with ≥1 night hospitalization in past month, n (%)	34 (2.3)	5 (3.9)	29 (2.1)	24 (2.4)	5 (5.7)	19 (2.1)

*AD* Alzheimer’s disease, *ADL* activities of daily living, *ER* emergency room, *HCRU* health care resource use, *iADL* instrumental ADL, *ZBI* Zarit Burden Interview

^a^Data are presented as mean (SD) or median (Q1–Q3), unless indicated otherwise. Percentages reported are for caregivers with data available for each specific variable. Missing data for the total AD dementia population (overall diabetes status) were *n* = 1 for all measures (for caregivers and patients) except ZBI score (*n* = 9). Missing data based on the population with mild/moderate AD dementia were *n* = 1 for all measures (for caregivers and patients) except ZBI score (*n* = 7)

^b^Diabetes status was unknown for one caregiver, thus *N* = 1494

**Fig. 2:**
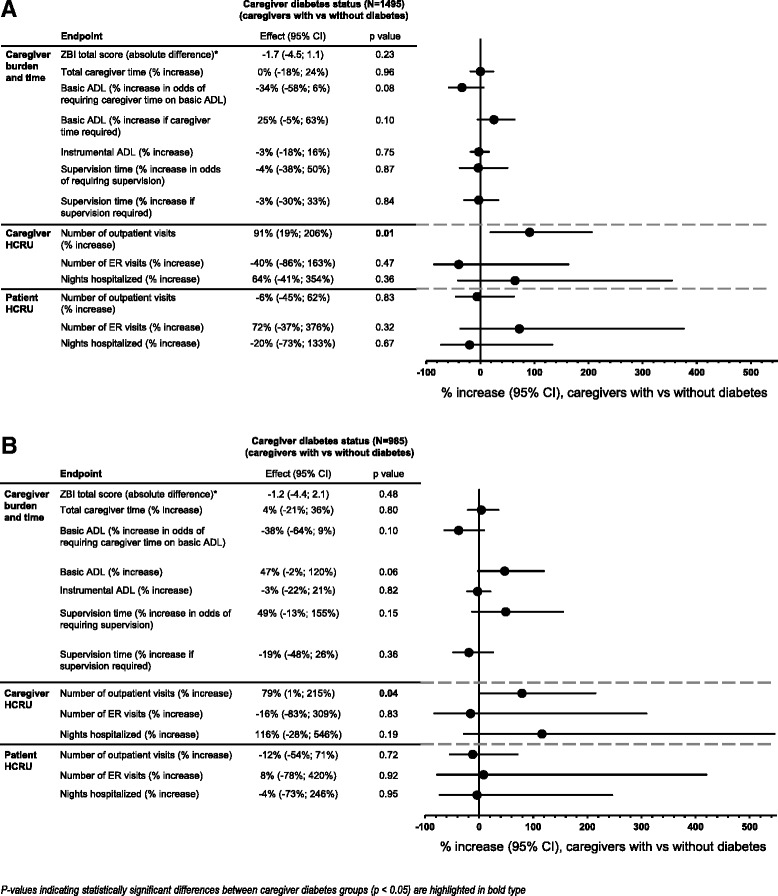
Caregiver burden/time, and caregiver and patient HCRU according to caregiver diabetes status. **a** Total AD dementia population. **b** Mild/moderate AD dementia population. AD Alzheimer’s disease, ADL activities of daily living, CI confidence interval, ER emergency room, HCRU health care resource use, ZBI Zarit Burden Interview. Data are presented as absolute difference or % increase with 95 % CIs for caregivers with versus without diabetes. HCRU measures are reported as the percentage increase in the odds of having outpatient or ER visits or of being hospitalized. *P*-values (obtained using likelihood ratio statistics for all measures except requirement for supervision and caregiver time on basic ADL, where only the Wald test was available) are for the comparison between caregiver diabetes groups. *As ZBI data are presented as absolute difference it was not possible to include this measure in the Forest plot with the effects of the other endpoints, which are presented as % increase

Caregiver diabetes status showed no clear or statistically significant effects on burden or caregiver time, although a trend toward lower burden in caregivers with versus without diabetes was observed (Fig. 2a).

#### Caregiver HCRU

Although 636 caregivers overall (42.6 %) had no outpatient visits (Table 4), caregivers with diabetes had a 91 % increase in the odds of having outpatient visits compared with caregivers without diabetes (*p* = 0.01; Fig. 2a). The odds of having ER visits or of being hospitalized did not differ between caregiver diabetes groups (Fig. 2a).

### Subgroup analysis: patient population with mild/moderate AD dementia only

To provide an assessment of the effect of AD dementia severity on the results, a secondary analysis was performed based on a subgroup of patients with mild/moderate AD dementia (*N* = 985). As seen in the main analysis, results for all burden/time measures were highly variable, both overall and within the different patient (Table 2) and caregiver (Table 4) diabetes groups. Patients with mild/moderate AD dementia were less likely to require supervision than those in the overall population with AD dementia (more than 50 % of patients with mild/moderate AD dementia had zero values for caregiver supervision hours [median supervision time was 0 h; Table 2]).

A total of 799 patients (81.2 %) and 574 caregivers (58.3 %) had one or more outpatient visit in the month before baseline (Tables 2 and 4). Less than 4 % of patients and caregivers overall had ER visits or hospitalizations (Tables 2 and 4).

Neither patient nor caregiver diabetes status showed clear or significant effects on burden, caregiver time, or caregiver or patient HCRU in analyses based on the patient population with mild/moderate AD dementia (Fig. 1b).

As seen for the total AD dementia severity population, caregivers with diabetes had a 79 % increase in the odds of having outpatient visits compared with caregivers without diabetes (*p* = 0.04), but the odds of having ER visits or of being hospitalized did not differ between caregiver diabetes groups (Fig. 2b).

### Sensitivity analyses

Sensitivity analyses showed that results were qualitatively unchanged by removing propensity scores and patient BMI, indicating that the conclusions of the main analyses were robust.

## Discussion

This analysis of a real-world population of community-dwelling patients with AD and their informal caregivers found that caregiver time spent on iADL and requirement for supervision were significantly higher for caregivers of patients with diabetes versus without diabetes. Although a consistent general increase in caregiver time on basic ADL, iADL, and total caregiver time was observed for caregivers of patients with diabetes, this effect was only statistically significant for iADL, possibly due to the variability within the dataset.

These results for caregiver time are in line with those of Langa et al. [24], who found that elderly Americans with diabetes required more time for informal care than those without diabetes, and indicate that this effect is also observed in patients with AD dementia who generally require more care than the general population of a similar age group.

The impact of patient diabetes status on caregiver burden was more evident for objective measures (caregiver time) than the subjective measure, ZBI score. A trend towards increased ZBI score was observed for caregivers of patients with versus without diabetes, but this small difference (+1.6) is unlikely to have significant real-life impact for the caregiver. This is in line with previous data suggesting that diabetes was not predictive of subjective burden in caregivers of community-dwelling frail elderly patients in Japan, measured using the Japanese version of the ZBI [23]. A comparative analysis also found caring for patients with cancer or dementia was associated with greater physical strain and emotional stress than caring for those with diabetes or for frail elderly people [36]. It is possible that the burden of caring for a person with AD dementia subsumes any added burden associated with diabetes. Feil et al. [22] reported a high level of subjective burden (according to the 4-item version of the ZBI) in caregivers of patients with both dementia and diabetes, but the sample size was small (*N* = 21) and the absence of a comparator group meant that it was not possible to assess the extent to which diabetes added to the burden of caring for someone with dementia alone.

Patient and caregiver HCRU was unaffected by patient diabetes status. However, HCRU data were based on the month before baseline only. Meaningful between-group differences may be observed over a longer period of time of prospective data capture, particularly on hospitalizations and ER visits which are expected to have a generally low base rate over a single month. Caregiver HCRU has previously been shown to be affected by several aspects of AD dementia [37], and our analysis provides new data regarding the effects of patient and caregiver diabetes in this population.

Analyses based on the subgroup of patients with mild/moderate AD dementia, found no consistent effects of patient diabetes status on burden, caregiver time, or caregiver or patient HCRU, suggesting that differences between the diabetes groups in the overall population were driven by the patients with moderately severe/severe AD dementia. This finding is consistent with our prior analysis showing that better patient functioning is associated with lower caregiver burden and fewer supervision hours in GERAS [21], although we did not adjust for functional status in our analysis. As patients with moderately severe/severe AD dementia would generally function at a lower level than patients with mild/moderate AD dementia, they would consequently be unlikely to be able to manage their own diabetes, thus increasing the time required from the caregiver. Caregivers of patients with mild/moderate AD dementia may also provide most care around mealtimes and bed times, which may coincide with any requirement for them to assist with diabetes treatment and blood glucose monitoring.

In the main analyses, time and burden were unaffected by caregiver diabetes status; again caregiver time was based on the month before baseline only (ZBI score reflected the subjective burden reported at the baseline visit). We had anticipated that caregivers with diabetes would report greater burden than those without diabetes, due to the added burden of managing their own diabetes along with the patient’s AD dementia; however, our results did not support this hypothesis. The reasons are unclear, but may be related to the small number of caregivers who had diabetes in this study. It is also possible that caregivers who have diabetes themselves and are already comfortable with managing their condition do not perceive the management of the patient’s diabetes as a significant additional burden; however, the study sample size was too small to allow a further subanalysis.

Caregivers with diabetes had increased odds of having outpatient visits for their own health care than caregivers without diabetes, which could be expected for people with chronic conditions. Other aspects of caregiver HCRU were not associated with caregiver diabetes status, regardless of the dementia severity of the AD patients under their care.

### Strengths

We used data from a large prospective observational study of community-dwelling patients with AD across a wide range of dementia severity groups in three European countries. This allowed a *post-hoc* exploratory analysis of the effects of diabetes on burden, as reported by caregivers of these patients, in a real-world setting.

To our knowledge, this is the first study to directly assess whether diabetes affects burden, time spent on caregiving, or HCRU in caregivers of patients with AD. Our findings add to those from other studies that have identified many factors that influence burden in caregivers of patients with AD [21, 38, 39]. Although we found no significant effect of patient diabetes on caregiver burden as assessed by ZBI, there was an increase in caregiver time requirement for patients with versus without diabetes.

Several standard measures of caregiver burden were assessed, including the well-recognized ZBI and caregiver time and HCRU were assessed using the standardized RUD instrument.

### Limitations

Although our study provides valuable new data, it has several limitations which provide a possible focus for future studies.

This was a *post-hoc* exploratory analysis, and a cross-sectional assessment of baseline data. Results may be different if a longitudinal perspective were to be used, and this would also allow an assessment of the effect of diabetes on caregiver burden and patient and caregiver HCRU over time. It would also identify whether the early differences in caregiver time measures translate to increased perceived burden recorded using the ZBI measure over a longer period.

HCRU information for the month prior to enrollment was based on caregivers’ report only and did not include information from medical records or claims data.

The self-reporting of diabetes status (or caregiver-reporting for some patients) in the GERAS study may limit the reliability of the information provided as the use of self-reported diabetes is generally considered to underestimate the actual prevalence, which includes a proportion of undiagnosed cases [40]. However, our patient diabetes prevalence of 12.6 % is in line with the 13–14 % seen in the largest studies reviewed by Bunn et al. [10] regarding diabetes prevalence in people with dementia, although reported prevalences ranged from 6 to 39 % [10]; diabetes prevalence data in this review were obtained from medical records and/or clinical examination. The older age of the patients in our study and/or presence of AD, may explain the expected higher diabetes prevalence in patients than in caregivers (12.6 and 8.5 %, respectively). Furthermore, we did not collect information on whether diabetes was type 1 or type 2, so are unable to confirm whether these proportions are in line with those seen typically for the different diabetes types. Links between cognition in AD dementia and both types of diabetes have been reported [41].

No information was obtained regarding the type or dosage of glucose-lowering medications or the level of diabetes control. It is possible that differences in burden/time existed regarding, for example, whether or not patients were receiving insulin and/or the amount of monitoring needed. Several aspects of diabetes care have been reported as being difficult to perform or supervise due to the patient’s dementia, particularly adherence to diabetes-related exercise regimes and dietary requirements [22]. Informal caregiver time was higher for elderly patients with diabetes who used insulin, compared with those taking no medication or oral medication in the study by Langa et al. [24]. However, although diabetes itself did not independently predict burden in the study by Hirakawa et al. [23], community-dwelling frail elderly patients with diabetes who were not taking medication required a higher degree of care compared with those taking diabetes medication or those without diabetes; these patients (with diabetes and no medication) were also more likely to suffer from hypertension and dementia. Patients with diabetes who were taking insulin used more transportation (and therefore required more time) than those on no or oral medication [23]. Effects of caregiver diabetes status on burden may also vary depending on their diabetes type, treatment, and severity.

Treating complications associated with diabetes may also increase caregiver burden. As data regarding specific diabetes-related complications were not collected in the GERAS study, we were also unable to assess whether such complications influenced our results. Increased emotional burden has previously been shown in caregivers of diabetes patients with foot ulcers [42].

### Suggestions for future research

This study provides new data on the overall effect of diabetes on subjective burden, time and HCRU in caregivers of patients with AD. Future research should include longitudinal analyses, assessing the change in these factors over time, and could consider the influence of various aspects of diabetes in order to inform appropriate support that could be given to patients and caregivers. Several diabetes-related issues (e.g., restricting intake of inappropriate foods, using a glucose meter, and providing assistance with oral medication) have been associated with objective burden in caregivers of patients with both schizophrenia and diabetes [43], and with subjective burden in caregivers of patients with both dementia and diabetes [22]. It is also possible that less time-consuming options for diabetes treatment and monitoring may offer a potential way to reduce burden, by reducing the time spent by the caregiver on iADL. This may include simplified treatment regimens, such as taking fewer medications, less frequent injections, or use of medical devices that allow for more automated monitoring and insulin administration. Such measures may also improve medication adherence and diabetes control in patients with dementia, who are less likely to receive their recommended annual monitoring for diabetes and may have poorer access to diabetes services [10, 44, 45]. Potential effects of other comorbidities in both patients and caregivers could also be examined: observational studies are likely to show greater heterogeneity in comorbidity data, compared with those from randomized clinical trials, and are therefore best suited to this type of analysis. The effect on caregiver health of caring for patients with diabetes and AD could also be investigated.

Further research, such as that identified, could provide opportunities to guide appropriate caregiver support strategies and policies. Improved support and education regarding diabetes care adjustments could potentially lessen caregiver burden [22]. As well as simplifying the patient’s medication as much as possible, the effects of additional help, such as assistance from professional caregivers or other family members to provide respite for the primary caregiver, also require consideration.

## Conclusions

Data from our study indicate that caregiver time spent on iADL and requirement for supervision was higher for caregivers of patients with AD with diabetes versus without diabetes. However, HCRU by both caregivers and patients in the month prior to study enrollment was unaffected by patient diabetes status.

Considering that this investigation was a cross-sectional analysis, future longitudinal studies assessing change in caregiver burden over time by patient diabetes status will help clarify the cumulative impact of diabetes and AD dementia on caregiver burden. Identifying factors that influence burden may also help to inform appropriate support that could be given to patients and caregivers.

### Ethics approval and consent to participate

National ethics committee approvals obtained:

- France: Commission Nationale de l’Iinformatique et des Libertes (CNIL) (reference EGY/DP/AR104863).
- Germany: Philipps University, Marburg, Fachbereich Medizin, Dekanat/Ethikkommission (reference 105/10).
- UK: NHS National Research Ethics Service, South West 5 REC (reference 10/H0107/43); Scotland A Research Ethics Committee (reference number 10/MRE00/63).

### Availability of data and materials

The dataset supporting the conclusions of this article are not publically available.

## Abbreviations

AD: Alzheimer’s disease
ADL: activities of daily living
ANCOVA: analysis of covariance
ANOVA: analysis of variance
BMI: body mass index
CI: confidence interval
ER: emergency room
EU: European Union
HCRU: health care resource use
iADL: Instrumental ADL
MMSE: Mini-Mental State Examination
Q: quartile
RUD: resource utilization in dementia
SD: standard deviation
USA: United States of America
ZBI: Zarit Burden Interview
